# Tissue‐specific reactions to positional discontinuities in the regenerating axolotl limb

**DOI:** 10.1002/reg2.35

**Published:** 2015-06-16

**Authors:** Malcolm Maden, Daima Avila, Molly Roy, Ashley W. Seifert

**Affiliations:** ^1^ Department of Biology and UF Genetics InstituteUniversity of FloridaGainesvilleFlorida32611USA; ^2^ Department of BiologyUniversity of KentuckyLexingtonKentucky40506USA

**Keywords:** Axolotl, chemotaxis, law of distal transformation, limb regeneration, supernumerary limbs

## Abstract

We investigated cellular contributions to intercalary regenerates and 180^o^ supernumerary limbs during axolotl limb regeneration using the cell autonomous green fluorescent protein marker and exchanged blastemas between white and green fluorescent protein animals. After distal blastemas were grafted to proximal levels tissues of the intercalary regenerate behaved independently with regard to the law of distal transformation; graft epidermis was replaced by stump epidermis, muscle‐derived cells, blood vessels, and Schwann cells of the distal blastema moved proximally to the stylopodium and cartilage and dermal cells conformed to the law. After 180^o^ rotation, blastemas showed contributions from stump tissues which failed to alter patterning of the blastema. Supernumerary limbs were composed of stump and graft tissues and extensive contributions of stump tissues generated inversions or duplications of polarity to produce limbs of mixed handedness. Tail skeletal muscle and cardiac muscle broke the law with cells derived from these tissues exhibiting an apparent anteroposterior polarity as they migrated to the anterior side of the blastema. We attribute this behavior to the possible presence of a chemotactic factor from the wound epidermis.

## Introduction

The concept of positional information (Wolpert 1969) has been thoroughly studied in regenerating systems such as the amphibian and insect limb to formulate laws that can explain how cells determine their position (relative to other cells) during morphogenesis (e.g., French *et al*. 1976; Meinhardt 1983). The elaboration of such laws is a prerequisite to a complete cellular and molecular understanding of organ regeneration and, as such, theoretical models for pattern formation represent important generalizations. Regenerating limbs provide the clearest realization of the nature of positional information. Amputation of the axolotl or newt limb through the humerus (mid‐upper arm level) results in the replacement of all tissues distal to the amputation plane (distal upper arm, lower arm and hand), whereas amputation through the radius and ulna (mid‐forearm level), despite being composed of the same panorama of differentiated tissues (e.g., muscle, cartilage, etc.), results in the replacement of only the distal forearm and hand. In addition, mis‐location of tissues or regeneration blastemas frequently results in extra structures or even complete supernumerary limbs forming from the amputation plane. It has been proposed that cells comprising the blastema possess molecular coding of their global position within the limb in order to correctly regenerate the precise structures which were removed by amputation.

The nature of this positioning system is routinely analyzed following positional mis‐location of tissues or blastemas to assess how different tissue confrontations can alter pattern formation. To analyze the proximodistal axis (shoulder to hand) distal blastemas are grafted to proximal stumps (or vice versa), and to analyze the anteroposterior axis and/or dorsoventral axis blastemas are grafted from left to right limbs or rotated 180^o^ on the same limb. The result of the former mis‐location—grafting a forearm blastema to an upper arm amputation level—is summarized as the law of distal transformation, a concept widely seen in regeneration studies in invertebrates and applied to the amphibian limb by Rose (1962). Following such an operation the disparity in level is recognized and the missing tissue, known as the intercalary regenerate, is filled in (Iten & Bryant 1975; Stocum 1975). Studies using grafts between black and white, diploid and triploid, or forelimb and hindlimb (Maden 1980a; Pescitelli & Stocum 1980) have suggested that the intercalary regenerate arises by distal transformation of cells from the proximal stump rather than the proximal transformation of cells from the distal blastema, hence preserving the law of distal transformation. However, these conclusions were only of a general nature because the origin of individual cells of the different tissues in the intercalary regenerate could not be precisely determined—triploid identification was only assessed on cartilage cells; color was determined by the presence or absence of melanophores in the dermis; forelimb or hindlimb was assessed by cartilage structure.

The result of the latter mis‐location—rotating blastemas or individual tissues such as skin or muscle—results in ectopic, supernumerary limbs (Lheureux 1972; Carlson 1975; Iten & Bryant 1975; Bryant & Iten 1976; Maden 1980b; Maden & Mustafa 1982). Using the triploid marking technique (which can only identify at best less than 50% of the cells in any section), only the cellular origin of supernumerary limbs following 180^o^ blastemal rotations has been examined (Maden & Mustafa 1984). Despite these limitations, these experiments established that such 180^o^ supernumeraries are chimeras, composed of both stump and graft tissues. Furthermore, tissue mixing within these chimeras causes structural changes in the supernumerary.

Since these experiments were performed, several transgenic axolotl lines have been created that ubiquitously express fluorescent proteins in a cell autonomous manner (Sobkow et al. 2006; Khattak et al. 2009). These lines have transformed the reliability of cell identification for positional studies. For example, using the green fluorescent protein (GFP) transgenic line, cell behavior of the cartilage/fibroblast lineage predicted from earlier transplant studies (Thornton 1938; Namenwirth 1974; Monaghan & Maden 2013) as well as lineage restriction of muscle‐derived cells revealed that blastema cells are not pluripotent but instead are composed of a lineage‐restricted population of progenitor cells with a cellular memory of their tissue derivation or embryonic germ layer origin (Kragl et al. 2009). With respect to positional information, it was found that Schwann cells did not adhere to the law of distal transformation since cells from the hand level when moved to the upper arm level could spread all along the limb in contrast to cartilage cells derived from the finger which homed back to distal levels (Kragl et al. 2009). Another recent revelation using transgenic lines found that myogenic cells appear to disobey the law of distal transformation because wrist blastemas can give rise to cells associated with muscle at all levels of the limb when transplanted to upper arm stumps (McCusker & Gardiner 2013; Nacu et al. 2013). Taken together, these findings suggest that the nature of positional information is cell autonomous and restricted to specific cell types.

Thus, surprising new information has been obtained with regard to the relevance of patterning models when classical grafting experiments are combined with contemporary transgenic lineage tracing techniques. In the work reported here we have performed the three grafting paradigms described above using ubiquitously expressing GFP tissues grafted to white hosts to investigate tissue‐specific positional commitment in (1) the proximodistal axis during intercalary regeneration, (2) the anteroposterior and dorsoventral axes after 180^o^ blastema rotations to generate supernumerary limbs, and (3) muscle tissue from different anatomical sources to the regenerating limb. We find a surprising disregard for positional restriction among most lineages in that muscle, Schwann cells, and endothelial cells all ignore the law of distal transformation during intercalary regeneration. Moreover, these cells types move extensively across the anteroposterior axis during supernumerary limb regeneration without changing pattern. The epidermis displays no characteristics consistent with positional information and, instead, migrates distally regardless of the underlying mesodermal tissue configuration. Only the fibroblast/cartilage lineage seems to be restricted in positional fate. When cardiac or skeletal muscle is grafted to regenerating limbs, muscle‐derived cells move both proximally into the stump and distally into the blastema, ultimately redifferentiating into multiple limb tissues. Regardless of the transplant position in the stump, there is a surprising positional effect on these cells as they enter the blastema in that all grafted cells migrate to the anterior side of the regenerating limb. Since neither the epidermis nor the muscle displayed positional properties in earlier experiments we attribute this behavior to a chemoattractant released by the wound epidermis and hypothesize that it is anteriorly located. Future experiments will be designed to identify this postulated molecule.

## Materials and Methods

All experiments were performed on axolotls (*Ambystoma mexicanum*) 8−13 cm in total length of white and GFP genotype. Animals were obtained from matings conducted in the Department of Biology, University of Florida. Animals were kept in 40% Holtfreter's solution and treated in accordance with the University of Florida's Institutional Animal Care and Use Committee's regulations.

Animals used for blastema transplants were first anesthetized in 0.1% MS‐222 and forelimbs or hindlimbs were amputated at either the wrist/foot level through the carpals/tarsals or at the mid‐humerus/femur level. Once regeneration had progressed to the late bud (Iten & Bryant 1973) or cone stage (Monaghan et al. 2014) they were carefully re‐amputated at the junction between the blastema and stump and transplanted ipsilaterally either to another limb whose blastema had just been removed (for blastemal rotations) or onto a freshly amputated limb (for distal to proximal shifts). The blood from the fresh amputation site served as an adhesive to attach the transplanted blastema and the animal was kept under moist paper towels for 10 min to allow the adhesion to take place before being returned to Holtfreter's. For cardiac and skeletal muscle grafts a donor GFP animal was euthanized and the relevant tissue was removed from the animal and inserted into a tunnel under the dorsal skin made with forceps on the recipient animal at the upper humerus/femur level. The small tunnel was allowed to heal for 1 day after which the limb of the recipient animal was amputated adjacent to the graft.

After blastemal exchanges or tissue grafts, animals were anesthetized at 2–3 day intervals and the limbs were imaged under a Leica fluorescent dissecting microscope to follow the behavior of the blastema or grafted tissue. When regeneration was complete, animals were anesthetized and the limbs were imaged and photographed under GFP fluorescence. Following imaging, the limbs were removed and fixed in 4% paraformaldehyde overnight at 4°C. Regenerates from distal to proximal shifts or regenerates and supernumeraries from 180^o^ rotations were then placed in 30% sucrose overnight and embedded in OCT for cryosectioning. Each limb was serially sectioned in its entirety at 25 μm and every section was collected and mounted in glycerol containing 1 in 10,000 Hoechst 33258. Every section was examined unstained under GFP fluorescence and analyzed for the presence of GFP cells. The tissue identity of GFP cells was recorded manually (drawings) and in photographic images. Regenerates from muscle grafts were dehydrated, embedded in paraffin wax, and sectioned at 10 μm for immunocytochemistry.

## Results

### Distal to proximal grafts, the proximodistal axis

To examine positional commitment in the proximodistal axis a group of GFP forelimbs and hindlimbs were amputated through the carpal/tarsal (wrist or foot) level and allowed to regenerate to the cone stage. The GFP blastemas were then removed and transplanted to corresponding white limb stumps that had been freshly amputated through the mid‐humerus/femur level thereby generating a large disparity in proximodistal level between the graft and stump (Fig. 1A). Eleven limbs regenerated with four (forelimbs) or five (hindlimbs) digits and the majority of the grafted tissue survived. These were photographed in whole mount, and then serially sectioned with every section examined to determine the location of transplanted GFP cells. The tissue identity of GFP cells was determined by morphology and location.

**Figure 1:**
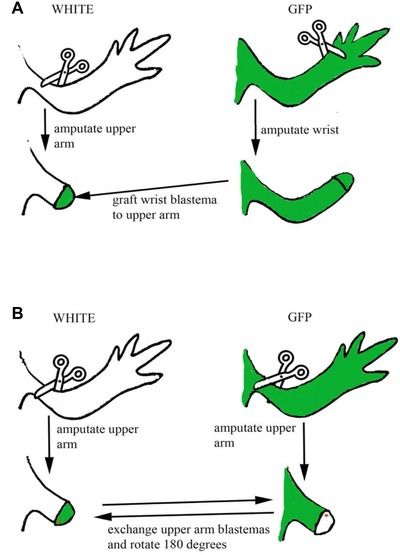
Summary of the surgical procedures undergone in the first two series of experiments. GFP animals are shown in green, white animals shown in white. (A) Distal wrist blastemas grafted ispilaterally to proximal mid‐stylopodial levels were used to investigate the contributions of stump and graft to the intercalary regenerate. (B) Mid‐stylopodial blastemas were grafted between GFP and white animals and rotated 180^o^ ipsilaterally during transplantation to generate maximal positional disparities in the circumferential axes.

According to the law of distal transformation we would expect a distal GFP blastema to generate all the tissues of the hand/foot whereas the host white stump should intercalate the missing distal humerus/femur tissue and all zeugopodial tissues. Only one case showed this result—a lack of any GFP tissue proximal to the hand—and this was one in which more than 50% of the graft had disappeared as only two digits were GFP. The remaining 10 cases all showed GFP tissue spreading proximally towards the elbow/knee, that is, throughout the zeugopodium, and furthermore half of these cases (5/10) showed GFP tissue at the humerus/femur level in gross contravention of the law of distal transformation. An example of a regenerate which looked superficially as if it corresponded with the expected behavior is shown in Figure 2A. However, upon sectioning, this regenerate showed GFP cells in the muscle of the zeugopodium (data not shown). An example of a regenerate that clearly contradicted the law is shown in Figure 2B. In this example, GFP cells spread proximally back to the humerus level. We obtained no examples (0/11) of completely white (proximal stump originating) intercalary regenerates.

**Figure 2:**
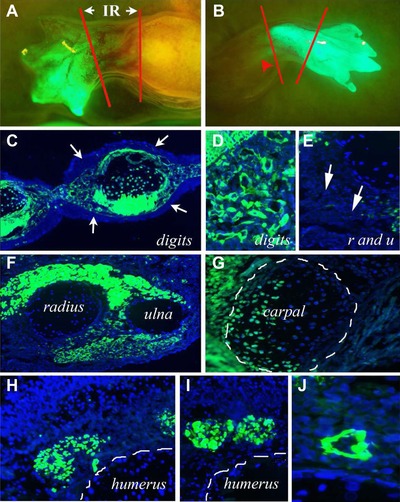
Intercalary regeneration. Micrographs show combined endogenous GFP fluorescence with Hoechst staining for nuclei. (A) The result of grafting a GFP wrist level blastema to a mid‐humerus level white stump. The stump is clearly white, the blastema had generated a clearly GFP wrist and hand and the intercalary regenerate (IR) between the two red lines looks mostly white and thus derived from the stump but also has some GFP cells within it. (B) Another wrist level GFP graft onto a mid‐humerus level white stump but in contrast to (A) the intercalary regenerate between the red lines looks all GFP and GFP cells are also visible proximal to the mid‐humerus amputation level (red arrowhead). (C) Section through the digits showing the GFP mesenchyme—muscles, cartilage, and connective tissue—and thus graft derived, surrounded by entirely white stump derived epidermis (white arrows). In all cases the proximal stump epidermis migrated distally over the graft and replaced the graft epidermis. (D) Close‐up of the dermis and connective tissue at the digit level showing it is composed of GFP cells and thus originated from the distal grafted blastema, in contrast to (E). (E) Close‐up of the dermis and connective tissue (white arrows) at the radius and ulna level (intercalary regenerate) showing it is composed of white cells and thus originated from the stump. (F) Section through the radius and ulna level of an intercalary regenerate showing it is composed of white radius and ulna (stump derived), GFP muscle (distal blastema derived), white dermis, connective tissue, and epidermis (stump derived). (G) Section through the cartilage of a digit (outlined in white lines) showing it is composed of both GFP cells (derived from the distal blastema) and white cells (derived from the proximal stump). (H), (I) Sections through the humerus level of a regenerate showing GFP muscles derived from the distal grafted blastema. (J) A blood vessel at the humerus level thus being derived from a distal grafted blastema.

#### Tissue specificity of the effect

Recent experiments have confirmed that blastemal cells retain cell lineage from their tissue of origin (Kragl et al. 2009) and these GFP blastemal transplants allowed us to examine specific tissue types at all limb levels to determine if some cell types obeyed the law of distal transformation while others did not.

*Epidermis*

In the majority of cases the epidermis of the grafted GFP distal blastema was completely replaced by host white epidermis even down to the level of the digits suggesting that the grafted blastemal epidermis is displaced by distal migration of the stump epidermis (Lheureux 1983). This resulted in GFP mesenchyme of the digits (graft) being covered by white (host) epidermis (Fig. 2C). Occasionally, graft epidermis was not entirely replaced, and in these cases a few GFP cells could be found in the epidermis (data not shown). This resulted in a seamless integration of the two genotypes rather than a sharp boundary with individual GFP cells surrounded by white epidermal cells.

*Dermis*

The dermis and its associated fibroblasts corresponded quite precisely with the law of distal transformation such that the grafted GFP distal blastemal cells generated the dermis in the carpal and digit region (Fig. 2D), whereas proximal to that level in the zeugopodium and stylopodium the dermis was white (Fig. 2E). However, individual tissues within the limb did not always correspond in the same fashion as the dermis (see below).

*Cartilage*

The majority of regenerates (6/10) showed cartilage which strictly adhered to the law of distal transformation; the distal humerus and radius and ulna were entirely composed of white cells (Fig. 2F) while the carpals were GFP+ (Fig. 2C). The remaining regenerates (4/10) contained GFP cartilage cells in the distal radius and ulna and we interpret this result to indicate that the precise level at which the grafted distal blastema was cut was proximal to the carpal level. However, no grafted GFP cells were seen in the cartilage more proximal than the distal radius and ulna level. For example, we never observed GFP+ cells in the humerus (Fig. 2H, I). Conversely, carpal and digit cartilages were not always composed entirely of grafted GFP cells as might be expected, but were sometimes composed of both graft and host cells in an apparently random fashion even to the extent that individual phalanges could be of mixed genotype (Fig. 2G).

*Muscle*

All of the regenerates had grafted GFP cells in the muscle of the intercalary regenerate proximal to the carpal level (Fig. 2C, F, H, I). At the zeugopodium level this resulted in clearly contrasting tissue origins—GFP grafted distal muscle (breaking the law of distal transformation) surrounding white host derived cartilage (conforming with the law of distal transformation) (Fig. 2F). Most significantly, 4 of the 10 regenerates had GFP muscle extending proximally past the elbow/knee and into the stylopodial level despite the cells originating from the carpal level (Fig. 2H, I). Muscle therefore clearly contradicted the law of distal transformation to a dramatic extent.

*Blood vessels and nerves*

Both blood vessels and nerves contained GFP+ cells at all limb levels and thus violated the law of distal transformation. In 4 out of 10 cases, both tissues showed examples of GFP cells located at the stylopodial level. When GFP+ nerves were detected they were associated with GFP+ muscles and thus it was likely that Schwann cells had migrated from distal levels (data not shown). GFP+ blood vessels most likely arose from endothelial cells that migrated from the distally transplanted blastema (Fig. 2J). Within the regenerated hand, sections also revealed that blood vessels were always GFP+ and so these must have originated from the blastema cells and not by ingression of white endothelium during revascularization from the stump. The tissue‐specific effects for all experiments described above are summarized in Figure 3.

**Figure 3:**
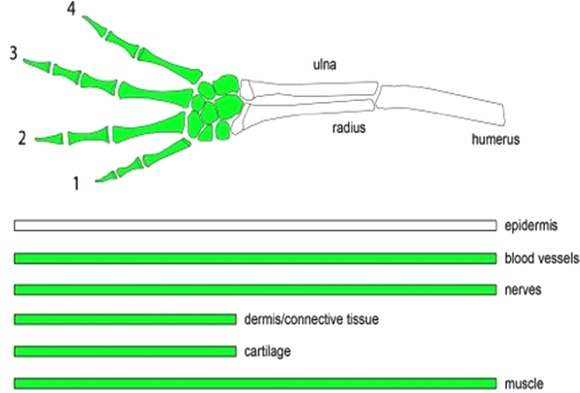
Summary of the results of the intercalary regeneration experiments testing the law of distal transformation. Above is a limb representing the proximodistal axis with GFP tissue grafted at the wrist level. Below are the individual tissues of the limb and their proximodistal distribution after distal grafting. Only cartilage and dermis conform with the law of distal transformation. Muscle, nerve, and blood vessels do not conform with the law and can spread proximally past their expected level; epidermis also does not recognize proximodistal levels as the stump epidermis is continually migrating distally.

### Ipsilateral 180^o^ blastemal rotations, the anteroposterior and dorsoventral axes

Having seen extensive mixing of cells in the proximodistal axis we next determined whether the same phenomenon occurs in the anteroposterior and dorsoventral axes during supernumerary limb production. Blastemas were generated on forelimbs and hindlimbs of white and GFP animals by amputation through the distal stylopodium just proximal to the elbow/knee. Regeneration blastemas were carefully re‐amputated at the junction between the stump and blastema and exchanged ipsilateral between white and GFP animals (Fig. 1B). During this exchange blastemas were rotated 180^o^ to reverse both the anteroposterior and dorsoventral axes (Fig. 1B). Four animals of each genotype were used giving a total of 32 rotated blastemas. Eight limbs exhibiting good, discrete supernumeraries (Fig. 4A) were selected for analysis. The remaining limbs produced either no supernumeraries (due to de‐rotation of the blastema) or a mass of digits that were impossible to analyze with confidence.

**Figure 4:**
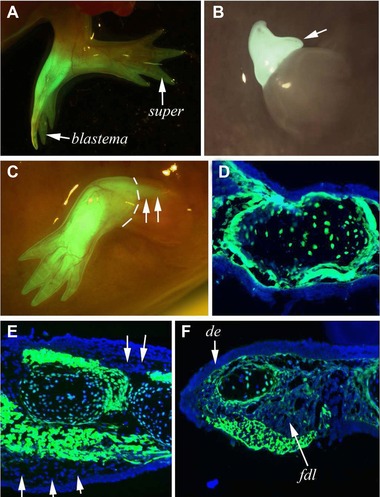
Supernumerary limbs and blastemas after 180^o^ blastemal rotation. (A) A limb with a rotated GFP blastema and a single supernumerary (super) which looks as though it is composed of half white/half GFP tissue. (B) A rotated GFP blastema showing the early appearance of a supernumerary (white arrow) at the blastema/stump junction. (C) A GFP blastema which completely de‐rotated and produced a complete limb in which the muscle moved proximally into the upper humerus level past the blastema/stump junction (marked with white dotted lines). (D)–(F) Sections through limb generated from the grafted and rotated GFP blastemas with varying contributions of stump cells. In (D) the mostly GFP (blastemal graft) mesodermal tissues are completely covered by white epidermis from the stump. In (E) there is a more significant contribution of stump (white) tissues making up not only the epidermis but also a large amount of dermis (white arrows). In (F) the dorsal epidermis is from the stump, there is a patch of blastemal derived GFP epidermis on the ventral side, and the flexor digitorum longis (*fdl*) is from the stump along with the dermis and connective tissue medial to the *fdl*.

The collective results of this series of experimental manipulations in terms of frequency of supernumeraries, their position, and digit numbers were the same as had been observed previously (Maden 1980b; Maden & Mustafa 1982). Out of eight limbs analyzed, half produced single supernumeraries and half produced doubles giving a total of 12 supernumeraries and eight blastemas for analysis. While the supernumeraries had the potential to originate from any position around the circumference, the vast majority arose from the posterior/ventral location. Five blastemas remained in their rotated position and generated supernumerary limbs that initially appeared as ectopic outgrowths (Fig. 4B), whereas three blastemas de‐rotated while still producing supernumeraries. The process of de‐rotation could not be directly observed during the subsequent observation period since no marking was performed on the blastemas. Instead it was inferred from the final position and cellular composition of the limbs and the fact that the process of supernumerary induction was readily followed and recorded at regular intervals (Fig. 4B). Some blastemas de‐rotated without producing supernumerary limbs and as a result produced normal limbs, albeit of a different genotype (i.e., GFP) (Fig. 4C). In these cases, a de‐rotated GFP blastema on a white stump produced a GFP+ limb perfectly congruous with the white stylopodial stump. This result highlights the observations made above on the lack of proximodistal positional information contained in muscle tissue because GFP+ muscle can clearly be seen proximal to the amputation plane running back to the shoulder (Fig. 4C, white arrows).

#### Cell contributions to the rotated blastema

Limbs generated from grafted blastemas and supernumerary limbs that were generated after 180^o^ rotations were serially sectioned and every section was examined to study the stump:graft cellular contributions. The rotated blastemas that developed into limbs revealed some surprising insights. As described above, the epidermis of the grafted blastema was consistently and almost entirely replaced by stump epidermis to generate GFP mesodermal tissues enveloped in white, stump epidermis (Fig. 4D). Stump tissues made major contributions to the mesodermal tissues of the developing blastema without causing any noticeable change in pattern. For example Figure 4E shows a GFP blastema that developed into a normal limb with white epidermis and an extensive contribution of white stump tissues to the dermis surrounding GFP+ muscle (Fig. 4E, white arrows). Figure 4F shows a limb with even more contribution of the stump tissues: here the ventral muscle (flexor digitorum longus [*fdl*]), dorsal epidermis, some dermis, and some cartilage cells are white (stump) whereas a section of the ventral epidermis, some dorsal dermis, the perichondrium, and some cartilage cells are GFP (graft). This analysis of the rotated blastema and the limb that it generates revealed that it develops from a variable contribution of both stump and graft tissues without any structural alterations.

#### Cell contributions to the supernumeraries

The handedness of supernumeraries corresponded to what has previously been described (Maden & Mustafa 1982), namely normal; mirror‐imaged in the dorsoventral axis; part normal/part mirror‐imaged; or part normal/part inverted. In contrast to the almost complete replacement of the grafted blastemal epidermis by the distal migration of stump epidermis, the supernumerary limbs maintained their relative contributions of stump and graft, but in a manner unrelated to the contributions of the mesodermal tissues. For example, the supernumerary pictured in Figure 4A maintained an almost 50:50 GFP+:white mix of epidermis but a much smaller contribution of GFP+ mesodermal tissues (Fig. 5A).

**Figure 5:**
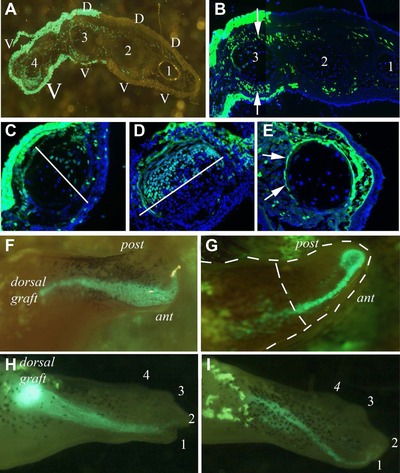
(A)−(E) Supernumerary limb structure after 180^o^ rotation. (A) Supernumerary with the first three digits of normal structure and the fourth of mirror‐imaged structure. The abnormality in the fourth digit is caused by the contribution of ventral GFP cells but it is clear that there is also a large contribution of GFP cells all across the limb which does not change the pattern of the other three digits. (B) Close‐up of digit 3 of the supernumerary in (A) showing a very significant contribution of GFP cells to the muscles which does not alter the pattern. (C) A digit of a supernumerary which is composed of GFP dorsal tissues and white ventral tissues yet the cartilage is anterior white and posterior GFP. A white line marks the GFP/stump tissue border in the cartilage which is at right angles to the rest of the mesodermal and epidermal tissues. (D) A complementary example of the composition of a supernumerary digit where the dorsal half of the cartilage is GFP and the ventral half is white. (E) A supernumerary digit which is double ventral caused by an influx of GFP cells to the upper part of the digit in which the GFP perichondrium encircles the cartilage made up of white cells. (F)−(I) Dedifferentiated cells from muscle grafts are drawn to the anterior side of the blastema in every case. (F) Tail skeletal muscle stream of GFP cells going to the anterior side from its dorsal grafted position. (G) Stream of GFP cells from a graft of cardiac atrium moving to the apical cap and doing a U‐turn. (H) Stream of GFP cells derived from a cardiac ventricle graft moving to digit 1 of the regenerate. (I) Stream of GFP cells from tail skeletal muscle moving to the digit 1 position.

As expected, the structure of the supernumerary was generally related to the contributions from the stump or blastema. Thus a part normal/part inverted limb was composed of, for the most part, half stump/half rotated blastema. However, that generality belies an extensive mixing of cells that did not cause patterning abnormalities and could clearly be seen when sections of the supernumeraries were studied in detail. For example the supernumerary in Figure 5A has three digits of normal dorsoventral polarity (digits 1−3) and one digit of double ventral polarity (digit 4). The abnormal muscle mass which makes the double ventral digit 4 is contributed entirely by GFP cells from the rotated blastema. Surprisingly, however, there is also an extensive contribution of GFP cells across the whole of the supernumerary into the muscle and dermis of digits 1−3 but without any change of polarity (Fig. 5B). Digit 3, for example, has a large number of GFP muscle cells in both the dorsal extensor digitorum longus (Fig. 5B, upper white arrow) and the ventral flexor digitorum longus (Fig. 5B, lower white arrow) as well as some perichondrial cells, yet there is no change in dorsoventral polarity.

The same mixing of cell types can be seen in other tissues, not just the muscle, although in the cartilage it is impossible to tell whether the pattern of a digit has been altered since they are circular in cross‐section with indistinguishable dorsoventral or anteroposterior axes. Nevertheless we observed extensive mixing of stump and graft tissues in the cartilage. Figure 5C shows a digit from a supernumerary limb with GFP contributing to the upper epidermis, dermis, and perichondrium, and white cells contributing to the lower epidermis, dermis, and perichondrium, yet a 50:50 contribution to the cartilage of the digit with a border which does not correspond to that of the soft tissues as it is along the dorsoventral axis (Fig. 5C, white line). In contrast, a 50:50 border of white and GFP cells runs along the anteroposterior axis in a digit from another supernumerary limb (Fig. 5D, white line). In Figure 5E is a digit from a supernumerary limb which has double ventral polarity caused by an influx of GFP connective tissue, dermis, and muscle cells to the upper part of the digit, and this influx also contributed to the GFP perichondrium enveloping a phalange composed entirely of white cartilage cells (Fig. 5E, white arrows). Based on these data, it appears that in the blastema and the supernumeraries there was a surprisingly extensive intermingling of stump and grafted cells.

### Proximodistal patterning with other muscle sources to the blastema

As described in the Introduction, a third type of tissue translocation that generates altered limb structures occurs when differentiated tissue is mis‐localized (e.g., skin or muscle) followed by limb amputation (Lheureux 1972; Carlson 1975). It is curious that muscle tissue should display this aspect of positional information when we have seen above that muscle myoblasts can spread throughout the proximodistal extent of the limb and can also contribute extensively across the anteroposterior axis without altering pattern. Perhaps this only happens when muscle tissue has already lost its differentiated state and generates blastemal cells (or more precisely generates Pax7+ satellite cells [Sandoval‐Guzman et al. 2014]), as when blastemas are grafted. A test of this idea would be to translocate fully differentiated muscle tissue (that is labeled) to the limb followed by amputation. To investigate this possibility, we transplanted fully differentiated GFP+ limb muscle with supposedly defined positional information (Carlson 1975) to test its ability to spread proximodistally or anteroposteriorly and to alter pattern in the regenerate through the production of supernumerary structures. Another possibility is that muscle cells migrate along the limb during regeneration because they have limb identity but not spatial identity across the three cardinal limb axes. In order to test this possibility, we used tail skeletal muscle and cardiac muscle to examine their positional behavior relative to limb skeletal muscle. Four samples each of limb (anterior stylopodial), tail (skeletal muscle), ventricle and atrium (cardiac muscle) from a GFP animal were grafted under the skin of the dorsal stylopodial region of uninjured white animals.

After amputating the limbs adjacent to the graft regular observations were made on the response of the grafts. In all cases cells broke away from the bulk of the graft and spread along the proximodistal extent of the regenerate displaying no proximodistal positional properties. GFP+ cells even migrated proximally from the site of grafting in the stump. Curiously, although transplanted GFP cells were grafted dorsally, all GFP cells localized to the *anterior* side of the regenerate. Thus, although we observed no proximodistal positional properties, these cells did reveal an apparent anteroposterior positional memory.

This was most clearly visualized during fragmentation of the grafted muscle as the blastema expanded. In every case cells from the muscle grafts (including cardiac muscle samples) were drawn into the regenerating limb and onto the anterior side (Fig. 5F–I). Although grafted dorsally in the limb, these cells exhibited a clear affinity for the anterior side of the blastema and regenerating limb. Figure 5F shows a graft of skeletal muscle whose cells migrated to the anterior side of a palette blastema, and Figure 5G shows a graft of atrium whose cells were drawn to the anterior side of a late cone blastema. In this second case, the stream of cells abruptly stopped at the distal tip and did not cross into the posterior compartment. As a result of this positional displacement, the grafted cells ended up mostly in digit 1—Figure 5H shows a graft of the ventricle ending up in digit 1 and Figure 5I shows a graft of skeletal muscle similarly located.

## Discussion

This study was designed with the intention of re‐investigating earlier experiments on positional interactions in the regenerating limb such as the analysis of proximodistal positional information by grafting distal blastemas to proximal levels (Iten & Bryant 1975; Stocum 1975; Maden 1980a; Pescitelli & Stocum 1980) or rotating blastemas 180^o^ on their stumps to analyze the relative contribution of stump and graft cells to the regenerates (Maden 1980b; Maden & Mustafa 1984). Some of these earlier experiments used grafts from black and white animals which only gave general impressions of contributions rather than precise analyses since the melanophores of black animals are present only around certain mesenchymal tissues and no information at all could be generated with regard to the epidermis. Triploid cells were also used as a source of grafted tissue, but in these cases a maximum of 50% of the cells (and usually a good deal fewer) could be reliably identified as triploid. Thus, cellular precision of the type presented in this work was not possible before the advent of ubiquitously expressing GFP axolotls. Cellular contributions from blastema and stump can now be determined with far greater certainty.

We have shown that following grafting of distal GFP blastemas to proximal white stumps the intercalary regenerate is composed of both graft and host cells depending on the tissue type. This is in contrast to earlier experiments which suggested that the intercalary regenerate was composed entirely of stump cells which had become more distal to fill in the gap in compliance with the law of distal transformation (Maden 1980a; Pescitelli & Stocum 1980). However, we showed here, as Nacu et al. (2013) recently demonstrated, that proximal muscle is routinely derived from distal blastemas and thus muscle cells apparently do not obey the law of distal transformation. We also observed here that blood vessel endothelium and Schwann cells of the nerve fibers behave in a similar fashion and can spread proximally up the limb from the distal blastema. Indeed this can also occur in a limb that simply receives a graft of muscle tissue (data not shown).

Neither does the epidermis display any proximodistal positional information because stump epidermis seems to be continually migrating distally to replace the epidermis of grafted blastemas. Indeed, Lheureux (1983) documented a similar phenomenon in unwounded epidermis, although the rate of distal movement in an unwounded limb was much slower. Based on our observations of epidermal replacement, epidermis has no relationship at all with the underlying mesodermal tissues and displays no proximodistal positional information. If indeed it did possess positional information or communicate such information it would have to continually re‐specify this information as it moved. In stark contrast to muscle and epidermal cells, connective tissue cells that gave rise to cartilage and dermis adhered to the law of distal transformation. Interestingly, these cells are derived from the lateral plate mesoderm lineage which has been proposed to pattern the developing limb (Christ et al. 1977; Grim & Wachtler 1991).

This tissue analysis could be used to identify potential regulators of positional information in the proximodistal axis. *Meis* genes, for example, are expressed according to the level of origin in the regenerating limb and are considered to be regulators of proximal identity since they are upregulated by a proximalizing dose of retinoic acid (RA) and their overexpression proximalizes the properties of distal blastemal cells (Mercarder et al. 2005). MEIS protein is expressed by connective tissue derived upper arm blastemas and not distal blastemas in support of the concept that connective tissue derived cells display positional information (Nacu et al. 2013). Myogenic‐derived blastemal cells and PAX7+ satellite cells, however, express MEIS protein at all proximodistal levels and these cells are not responsive to proximodistal positional information suggesting that the presence of MEIS protein is not the sole proximal determinant for muscle cells (Nacu et al. 2013). We might expect cells that are not responsive to proximodistal patterning not to express MEIS proteins, but muscle cells are clearly not behaving in this manner. In contrast, however, Schwann cells which as we have seen above break the law of distal transformation do not express MEIS protein (Kragl et al. 2009).

Another molecule crucially involved in proximodistal positioning is Prod1 (da Silva et al. 2002), a GPI‐linked cell surface molecule expressed at higher levels in proximal blastemas than distal blastemas and when overexpressed in distal blastemas allows these cells to break the law of distal transformation and contribute to a more proximal level than expected (Echeverri & Tanaka 2005). Interestingly, the Prod1 promoter contains MEIS binding sites (Shaikh et al. 2011). It is expressed in the normal limb in a graded fashion with higher levels proximally (Kumar et al. 2007), but its tissue‐specific expression is not known apart from its suggested presence in Schwann cells. If indeed Prod1 plays a crucial role in proximodistal patterning, we would predict on the basis of the above results that it should be expressed in connective tissue fibroblasts, including dermal fibroblasts, cartilage precursors, and perichondrial cells, but not in myoblasts, Schwann cells, endothelial cells, and epidermal cells.

After 180^o^ rotation of blastemas, supernumeraries that were generated were of the same range of abnormal structures that had been seen before—part normal/part mirror‐imaged, part normal/part inverted (Maden & Mustafa 1982)—and we confirmed that the region of the supernumerary that was abnormally patterned was contributed by the stump. However, we also observed a widespread mixing of some cells across the supernumerary limbs that did not alter the pattern of that region generating mosaic cartilage, dermis, and muscle. This also occurred in the grafted blastema which could contain an extensive contribution of cells from the stump without changing the pattern. Presumably there is a threshold number of cells that originate from a different position in the limb and which can contribute to a structure before the patterning is changed by their presence. In terms of muscle structure this must be at least 50% of the cells because some muscles were seen with very significant contributions of “foreign” cells yet with normal pattern. We also observed muscle cells migrating proximally from the blastema to contribute to muscles above the amputation plane demonstrating the lack of proximodistal positional information in myoblasts. The epidermis of these blastemal rotation experiments, as in the case of proximodistal shifts, showed no positional behavior and could mingle across the anteroposterior axis as single cells, as larger groups of cells, or remain with fairly sharp borders while undergoing its primary behavior—migrating distally.

In an effort to investigate whether other sources of muscle display positional information in the limb, grafting cardiac muscle from both the atria and ventricles or skeletal muscle from the tail or the limb revealed a very striking behavior. In all cases, dorsally grafted muscle released cells which streamed into the blastema to the *anterior* side of the regenerating limb. These cells contributed to a variety of tissues in the regenerate—dermis, skeletal muscle, cartilage, ligament, axon sheaths, blood vessels, but all in the region of digit 1 on the anterior side. This streaming behavior is reminiscent of the ingression of cells from the somite to the limb bud during limb development under the control of hepatocyte growth factor expressed by limb bud mesenchymal cells (Scaal et al. 1999) and we may be observing a recapitulation of this activity during regeneration. Because intact pieces of muscle tissue were grafted into the limb, and this tissue contains fibroblasts as well as myofibers, the precise lineage of cells that showed the observed streaming behavior could not be conclusively determined. However, additional experiments revealed that this behavior was not observed following grafting of neural tissues (data not shown). One possible explanation of the behavior is the presence of chemotactic factors released by the apical cap since this is the final location of the distally migrating cells (Fig. 5G). This could explain why dedifferentiated cells move distally into the blastema during the normal process of regeneration, but to explain this anterior directed behavior the chemotactic factor would have to be expressed only on the anterior of the apical cap. The apical cap is known to express components of the RA, Wnt, insulin‐like growth factor and bone morphogenetic protein pathways (Monaghan et al. 2012) and several fibroblast growth factors (FGFs) including *Fgf‐1*, *Fgf‐2* and *Fgf‐8* (Boilly et al. 1991; Mullen et al. 1996; Christensen et al. 2001; Han et al. 2001). All of these are secreted signaling molecules and two of them display chemotactic properties in other systems: RA for neurites (Maden et al. 1998) and FGF4 in the chick limb bud (Li & Muneoka 1999). Furthermore both FGF4 and FGF2 are chemotactic for myogenic cells, the very cells which were used here (Itoh et al. 1996; Webb et al. 1997). However, in addition they would have to be expressed asymmetrically on the anterior side of the apical cap, a phenomenon not previously described in the regeneration literature. Strikingly, however, asymmetrical expression of signaling molecules occurs in the apical ectodermal ridge of the developing chick limb bud where *Fgf4* and *Bmp2* are expressed on the posterior side, the apical ectodermal ridge itself is an asymmetrical structure with taller epithelium on the posterior side (Niswander et al. 1994; Niswander & Martin 1992; Laufer et al. 1994; Pizette & Niswander 1999), and one of the functions of FGF4 is as a chemotactic factor drawing proliferating limb bud cells distally to elongate the developing bud (Li & Muneoka 1999). This scenario is remarkably similar to what may be occurring in the regenerating limb, albeit of opposite polarity, and we propose in future experiments to investigate the phenomenon of chemotaxis of blastemal cells by the apical cap. Further experiments exploring this phenomenon should help reveal how the wound epidermis directs blastema formation and limb patterning during regeneration.

## References

[reg235-bib-0001] Boilly, B. , Cavanaugh, K.P. , Thomas, D. , Hondermarck, H. , Bryant, S.V. & Bradshaw, R.A. (1991). Acidic fibroblast growth factor is present in regenerating limb blastemas of axolotls and binds specifically to blastema tissues. Dev Biol, 145, 302–310. 2040374 10.1016/0012-1606(91)90128-p

[reg235-bib-0002] Bryant, S.V. & Iten, L.E. (1976). Supernumerary limbs in amphibians: experimental production in *Notophthalmus viridescens* and a new interpretation of their formation. Dev Biol, 50, 212–234. 1269829 10.1016/0012-1606(76)90079-8

[reg235-bib-0003] Carlson, B.M. (1975). Multiple regeneration from axolotl limb stumps bearing cross‐transplanted minced muscle regenerates. Dev Biol, 454, 203–208. 1181217 10.1016/0012-1606(75)90255-9

[reg235-bib-0004] Christ, B. , Jacob, H.J. & Jacob, M. (1977). Experimental analysis of the origin of the wing musculature in avian embryos. Anat Embryol, 150, 171–186. 857700 10.1007/BF00316649

[reg235-bib-0005] Christensen, R.N. , Weinstein, M. & Tassava, R.A. (2001). Expression of fibroblast growth factors in regenerating limbs of Ambystoma: cloning and semi‐quantitative RT‐PCR expression studies. J Exp Zool, 290, 529–540. 11555861 10.1002/jez.1097

[reg235-bib-0007] Echeverri, K. & Tanaka, E.M. (2005). Proximodistal patterning during limb regeneration. Dev Biol, 279, 391–401. 15733667 10.1016/j.ydbio.2004.12.029

[reg235-bib-0008] French, V. , Bryant, P.J. & Bryant, S.V. (1976). Pattern regulation in epimorphic fields. Science, 193, 960–981. 10.1126/science.948762 948762

[reg235-bib-0009] Grim, M. & Wachtler, F. (1991). Muscle morphogenesis in the absence of myogenic cells. Anat Embryol, 183, 67–70. 2053710 10.1007/BF00185836

[reg235-bib-0010] Han, M.J. , An, J.Y. & Kim, W.S. (2001). Expression patterns of Fgf‐8 during development and limb regeneration of the axolotl. Dev Dynam, 220, 40–48. 10.1002/1097-0177(2000)9999:9999<::AID-DVDY1085>3.0.CO;2-8 11146506

[reg235-bib-0011] Iten, L.E. & Bryant, S.V. (1973). Forelimb regeneration from different levels of amputation in the newt, *Notophthalmus viridescens*: length, rate and stages. Wilhelm Roux’ Arch, 173, 263–282. 10.1007/BF00575834 28304797

[reg235-bib-0012] Iten, L.E. & Bryant, S.V. (1975). The interaction between the blastema and stump in the establishment of the anterior−posterior and proximal−distal organization of the limb regenerate. Dev Biol, 44, 119–147. 1132582 10.1016/0012-1606(75)90381-4

[reg235-bib-0013] Itoh, N. , Mima, T. & Mikawa, T. (1996). Loss of fibroblast growth factor receptors is necessary for terminal differentiation of embryonic limb muscle. Development, 122, 291–300. 8565841 10.1242/dev.122.1.291

[reg235-bib-0014] Khattak, S. , Richter, T. & Tanaka, E.M. (2009). Generation of transgenic axolotls (*Ambystoma mexicanum*). Cold Spring Harb Protoc, 8: prot5264. 10.1101/pdb.prot5264 20147240

[reg235-bib-0015] Kragl, M. , Knapp, D. , Nacu, E. , Khattak, S. , Maden, M. , Epperlein, H.H. , et al. (2009). Cells keep a memory of their tissue origin during axolotl limb regeneration. Nature, 460, 60–65. 19571878 10.1038/nature08152

[reg235-bib-0016] Kumar, A. , Gates, P.B. & Brockes, J.P. (2007). Positional identity of adult stem cells in salamander limb regeneration. CR Biologies, 330, 485–490. 10.1016/j.crvi.2007.01.006 17631442

[reg235-bib-0017] Laufer, E , Nelson, C.E. , Johnson, R.L. , Morgan, B. A. & Tabin, C. (1994). Sonic hedgehog and Fgf‐4 act through a signaling cascade and feedback loop to integrate growth and patterning of the developing limb bud. Cell, 79, 993–1003. 8001146 10.1016/0092-8674(94)90030-2

[reg235-bib-0018] Lheureux, E. (1972). Contribution a l'etude du role de la peau et des tissues axiaux du member dans la declenchement de morphogenesis regeneratrices abnormales chez le triton Pleurodeles waltlii Michah. Ann Embryol Morphogen, 5, 165–178.

[reg235-bib-0019] Lheureux, E. (1983). Replacement of irradiated epidermis by migration of non‐irradiated epidermis in the newt limb: the necessity of healthy epidermis for regeneration. J Embryol Exp Morphol, 76, 217–234. 6355358

[reg235-bib-0020] Li, S. & Muneoka, K. (1999). Cell migration and chick limb development: chemotactic action of FGF‐4 and the AER. Dev Biol, 211, 335–47. 10395792 10.1006/dbio.1999.9317

[reg235-bib-0021] Maden, M. (1980a). Intercalary regeneration in the amphibian limb and the rule of distal transformation. J Embryol Exp Morphol, 56, 201–209. 7400743

[reg235-bib-0022] Maden, M. (1980b). Structure of supernumerary limbs. Nature, 287, 803–805.

[reg235-bib-0023] Maden, M. & Mustafa, K. (1982). The structure of 180^o^ supernumerary limbs and a hypothesis of their formation. Dev Biol, 93, 257–265. 7128935 10.1016/0012-1606(82)90257-3

[reg235-bib-0024] Maden, M. & Mustafa, K. (1984). The cellular contributions of blastema and stump to 180^o^ supernumerary limbs in the axolotl. J Embryol Exp Morphol, 84, 233–253. 6533251

[reg235-bib-0025] Maden, M. , Keen, G. & Jones, G.E. (1998). Retinoic acid as a chemotactic molecule in neuronal development. Int J Dev Neurosci, 16, 317–322. 9829167 10.1016/s0736-5748(98)00046-x

[reg235-bib-0028] McCusker, C.D. & Gardiner, D.M. (2013). Positional information is reprogrammed in blastema cells of the regenerating limb of the axolotl (*Ambystoma mexicanum*). PloS One 8:e77064. 24086768 10.1371/journal.pone.0077064 PMC3785456

[reg235-bib-0026] Meinhardt, H. (1983). A boundary model for pattern formation in vertebrate limbs. J Embryol Exp Morphol, 76, 115–137. 6631316

[reg235-bib-0027] Mercader, N. , Tanaka, E.M. & Torres, M. (2005). Proximodistal identity during vertebrate limb regeneration is regulated by Meis homeodomain proteins. Development, 132, 4131–4142. 16107473 10.1242/dev.01976

[reg235-bib-0029] Monaghan, J.R. & Maden, M. (2013). Cellular plasticity during vertebrate appendage regeneration. Curr Topics Microbiol Immunol, 367, 53–74. 10.1007/82_2012_288 23239234

[reg235-bib-0030] Monaghan, J. , Athippozy, A. , Seifert, A.W. , Putta, S. , Stromberg, A. , Maden, M. , et al. (2012). Gene expression patterns specific to the regenerating limb of the Mexican axolotl. Biol Open, 1, 937–948. 23213371 10.1242/bio.20121594 PMC3507169

[reg235-bib-0031] Monaghan, J.R. , Stier, A.C. , Michonneau, F. , Smith, M.D. , Pasch, B. , Maden, M. , et al. (2014). Experimentally induced metamorphosis in axolotls reduces regenerative rate and fidelity. Regeneration 1, 2–14. 10.1002/reg2.8 PMC4895291 27499857

[reg235-bib-0032] Mullen, L.M. , Bryant, S.V. , Torok, M.A. , Blumberg, B. & Gardiner, D.M. (1996). Nerve dependency of regeneration: the role of Distal‐less and FGF signaling in amphibian limb regeneration. Development, 122, 3487–3497. 8951064 10.1242/dev.122.11.3487

[reg235-bib-0033] Nacu, E. , Glausch, M. , Le, H.Q. , Damanik, F.F.R. , Schuez, M. , Knapp, D. , et al. (2013). Connective tissue cells, but not muscle cells, are involved in establishing the proximo‐distal outcome of limb regeneration in the axolotl. Development, 140, 513–518. 23293283 10.1242/dev.081752

[reg235-bib-0034] Namenwirth, M. (1974). The inheritance of cell differentiation during limb regeneration in the axolotl. Dev Biol, 41, 42–56. 4140121 10.1016/0012-1606(74)90281-4

[reg235-bib-0035] Niswander, L. & Martin, G.R. (1992). Fgf‐4 expression during gastrulation, myogenesis, limb and tooth development in the mouse. Development, 114, 755–768. 1618140 10.1242/dev.114.3.755

[reg235-bib-0036] Niswander, L. , Jeffrey, S. , Martin, G.R. & Tickle, C. (1994). A positive feedback loop coordinates growth and patterning in the vertebrate limb. Nature, 371, 609–612. 7935794 10.1038/371609a0

[reg235-bib-0037] Pescitelli, M.J. & Stocum, D.L. (1980). The origin of skeletal structures during intercalary regeneration of larval Ambystoma limbs. Dev Biol, 79, 255–275. 7000577 10.1016/0012-1606(80)90115-3

[reg235-bib-0038] Pizette, S. & Niswander, L. (1999). BMPs negatively regulate structure and function of the limb apical ectodermal ridge. Development, 126, 883–94. 9927590 10.1242/dev.126.5.883

[reg235-bib-0039] Rose, S.M. (1962). Tissue‐arc control of regeneration in the amphibian limb In: Regeneration: 20th Growth Symposium, ed. Rudnick D. Ronald Press, New York, pp. 153–176.

[reg235-bib-0040] Sandoval‐Guzman, T. , Wang, H. , Khattak, S. , Schuez, M. , Roensch, K. , Nacu, E. , et al. (2014). Fundamental differences in dedifferentiation and stem cell recruitment during skeletal muscle regeneration in two salamander species. Cell Stem Cell, 14, 1–14. 24268695 10.1016/j.stem.2013.11.007

[reg235-bib-0041] Scaal, M. , Bonafede, A. , Dathe, V. , Sachs, M. , Cann, G. , Christ, B. , et al. (1999). SF/HGF is a mediator between limb patterning and muscle development. Development, 126, 4885–4893. 10518504 10.1242/dev.126.21.4885

[reg235-bib-0042] Shaikh, N. , Gates, P.B. & Brockes, J.P. (2011). The Meis homeoprotein regulates the axolotl Prod1 promoter during limb regeneration. Gene, 484, 69–74. 21684325 10.1016/j.gene.2011.06.003 PMC3152680

[reg235-bib-0006] da Silva, S.M. , Gates, P.B. & Brockes, J.P. (2002). The newt ortholog of CD59 is implicated in proximodistal identity during amphibian limb regeneration. Dev Cell, 3, 547–555. 12408806 10.1016/s1534-5807(02)00288-5

[reg235-bib-0043] Sobkow, L. , Epperlein, H.H. , Herklotz, S. , Straube, W.L. & Tanaka, E.M. (2006). A germline GFP transgenic axolotl and its use to track cell fate: dual origin of the fin mesenchyme during development and the fate of blood cells during regeneration. Dev Biol, 290, 386–397. 16387293 10.1016/j.ydbio.2005.11.037

[reg235-bib-0044] Stocum, D.L. (1975). Regulation after proximal or distal transposition of limb regeneration blastemas and determination of the proximal boundary of the regenerate. Dev Biol, 45, 12–116. 10.1016/0012-1606(75)90246-8 1181213

[reg235-bib-0045] Thornton, C.S. (1938). The histogenesis of the regenerating fore limb of larval Amblystoma after exarticulation of the humerus. J Morph, 62, 219–241.

[reg235-bib-0046] Webb, S.E. , Lee, K.K. , Tang, M.K. & Ede, D.A. (1997). Fibroblast growth factors 2 and 4 stimulate migration of mouse embryonic limb myogenic cells. Dev Dyn, 209, 206–216. 9186055 10.1002/(SICI)1097-0177(199706)209:2<206::AID-AJA6>3.0.CO;2-M

[reg235-bib-0047] Wolpert, L. (1969). Positional information and the spatial pattern of cellular differentiation. J Theor Biol, 5, 1–47. 4390734 10.1016/s0022-5193(69)80016-0

